# A Personal Critical Analysis of the Foundation Programme Curriculum

**DOI:** 10.15694/mep.2019.000083.1

**Published:** 2019-04-15

**Authors:** Hassaan Waqar

**Affiliations:** St Helens and Knowsley Teaching Hospitals NHS Trust – Lead Employer

**Keywords:** Foundation Programme, Foundation Programme Curriculum, Curriculum Models, Curriculum Aims, Assessment, Quality Assurance

## Abstract

This article was migrated. The article was marked as recommended.

The Foundation Programme (FP) is a two-year period where medical graduates are able to learn in the workplace in the United Kingdom. The Foundation Programme Curriculum (FPC) is designed to imbue trainees with the knowledge, skills and attitudes to be able to enter into speciality training. The reasons for the introduction of the FP and its curriculum will be discussed. Additionally, curricular aims and models pertaining to the curriculum of the FP will be discussed as will the relevance of the hidden curriculum to the FPC. The assessment strategies and quality assurance methods relating to the FPC will be also be discussed. The author has been a recent FP trainee and uses education theory in his assessment of its curriculum. In this personal critical analysis, I aim to review the curriculum of the FP in order to determine if it is an appropriate vehicle to transmit the necessary knowledge, skills and attributes for trainees to enter the next stage of their training.

## Introduction

A curriculum was defined by R.M. Harden as:

“.. more than just a syllabus or a statement of content. A curriculum is about what should happen in a teaching programme - about the intention of teachers and the way they make this happen” (Dent and Harden, 2009).

The FP is a two-year period where medical graduates are able to learn in the workplace in the United Kingdom. This entails application of the knowledge that they have gained at medical school in order to develop their clinical and professional capabilities as a doctor. The FP enables those who successfully complete it to go on to further training. The FPC is designed to imbue trainees with the knowledge, skills and attitudes to be able to enter into this next phase of their training. The FPC was designed in response to legislative change and applies educational theory in order to deliver an effective curriculum. The FPC is regulated in part by stakeholders in order to match the high expectation that the public have of all doctors. Assessment tools are used to ensure that trainees have met the minimum level of competence in multiple faculties in order to progress to the next stage of their training. Curricula are designed to meet particular aims and there are various models of curriculum design which can be used to achieve these objectives. There are various quality assurance processes involved in maintaining and refining a curriculum in order to ensure its effectiveness in light of changes to practice. I aim to explore the above themes and concepts and relevant educational, social and political influences on the FPC. This is in order to determine if the FPC is an appropriate vehicle to transmit the necessary knowledge, skills and attitudes for trainees to enter the next stage of their training.

## Why were the FP and the FPC introduced?

Prior to the introduction of the FP, postgraduate medical training suffered from flaws such as poorly planned training, insufficient supervision and assessment. In addition, training was thought to be inflexible and suffered from poor job structure, with unstandardised appointment procedures which were non competency dependant. In response to this, the Department of Health commissioned a review by the Chief Medical Officer (CMO) in 2002 that highlighted the above concerns and recommended a restructuring of postgraduate medical training by focusing on the Senior House Officer (SHO) grade. The SHO grade, at the time, was all doctors who had completed at least one year of training after graduation, known as the Pre-registration House Officer grade (PRHO), but who had not yet progressed to a Speciality Registrar position (Donaldson, 2002).

The consultation document recommended the creation of a two-year FP which would include the PRHO grade and enable doctors to gain core skills early on in their first two years after graduation from medical school. It also encouraged the creation of a curriculum based programme to address the unstructured SHO training pathway that existed at the time. The consultation document does not appear to have been well received by the doctors’ union the British Medical Association (BMA). The BMA suggested, according to Madden and Madden (2007) that some of the recommendations were wide ranging, possibly going beyond the CMO’s remit. Responses to the consultation were however, mostly positive as Madden and Madden (2007) recall, despite one responder suggesting that there was an ulterior motive which aimed to introduce change without proper consultation.

This was followed up by Department of Health policy which outlined the two-year programme consisting of rotations in various clinical specialities, at the end of which trainees would be eligible to enter speciality training. The first year of the programme, known as Foundation Year One (FY1) was similar to the existing PRHO grade, after which trainees gained full registration with the General Medical Council (GMC). The SHO grade was split into Foundation Year Two (FY2) grade and various Core Training (CT) and Speciality Training (ST) grades (Department of Health, 2004). This policy document required the establishment of a curriculum for the FP in order to determine if trainees had met a pre-defined number of competencies. The FPC was established based upon specific principles, such as defining the competencies being assessed and the method of their assessment. The first iteration of the FPC (The Foundation Programme Committee, 2005) was produced with the assistance of the Department of Health lead by the same CMO who wrote the consultation document mentioned earlier. Whilst it is unclear the extent to which the FPC was politicised, given the CMO having direct input into its first iteration, it appears that some of the changes may have been politically driven following the CMO’s initial consultation document.

Additionally, the FP itself was set up to meet the legal requirements of the GMC and had to adapt to fit with the European Working Time Directive (EWTD) which limited the number of hours a doctor could work in an average week as reported in the literature by The Lancet (2005). The EWTD had a profound impact on the working lives of Foundation Year trainees as it affected rostering of junior doctors. The 48 average working week as legislated by the EWTD was analysed by Goddard, Hodgson and Newbery (2010) who found that, possibly as an indirect result of this legislation, Foundation Year doctors were the most senior doctor on call at night in certain hospitals. This appears to be contrary to one the reasons that the FP and by extension its curriculum was introduced, which was to ensure adequate supervision of trainees. This highlights that whilst the FPC may have been designed to be an ideal curriculum, its delivery has to some degree been hampered by wide ranging political changes.

## Curriculum models

The FP could be thought of as a spiral curriculum as described by Harden, Davis and Crosby (1997). In this curriculum model, new content is linked to prior learning and previously learnt topics are revisited in order to reinforce prior learning. The FPC divides expected learning into two years, however competencies are revisited and those gained in the second year build upon those acquired in FY1. Additionally, this model has accounts for certain content being more challenging than others and that the competence of students increases. The FPC encourages FY2 trainees to take on more responsibility in managing patients’ medical problems, possibly as they now have full GMC registration and they have more experience, and so are able to make more senior decisions compared to a FY1 junior doctor.

Harden (2000) describes a ladder approach to integration of content in a curriculum. This integration ladder lists 11 points on a continuum from isolation progressing through harmonisation to end with a transdisciplinary approach to teaching and learning. The ladder approach describes the extent to which individual sections of teaching and learning interact with each other, from no interaction (isolation) to between different disciplines (multi-disciplinary) and ending in interaction occurring in the students’ minds (trans-disciplinary). It is difficult to assess how integrated the FPC is and indeed its position on this ladder.

The curriculum can be thought of as being integrated as shown by the method of its assessment. The curriculum requires trainees to evidence that they are acquiring the required competencies using an electronic portfolio (e-portfolio). The e-portfolio lists specific core procedures that a Foundation Year trainee has to have evidence of competing in order to pass the year at their Annual Review of Competence Progression (ARCP). These procedures, such as interpretation of an ECG requires parallel knowledge of clinical sciences such as physiology (to understand how an ECG trace is produced) and cardiology (to understand what each section of the ECG trace means). As completion of these competencies is mandatory to pass the ARCP, it indirectly leads to an integrated curriculum.

Regarding the position of the curriculum on the integration ladder, the curriculum, if viewed as a syllabus, does not explicitly require trainees to have teaching by other health professionals. If this did occur, then it would meet at least the multi-disciplinary stage of the integration ladder. However, in practice, some teaching may be delivered by nurses and other allied health professionals, and this may be assessed through a trainee’s e-portfolio by senior clinicians at the ARCP. This ambiguity, amongst others, mean that it is difficult to accurately pinpoint the degree to which the curriculum is integrated if viewed through the lens of a specific ladder. However, given that the FPC places a large emphasis on the trainee’s ability as a self-reflective learner, it may be that the curriculum is at the upper end of the integration ladder.

Malik and Malik (2011) recognise that integration can be both vertical and horizontal in nature. Vertical integration is described as being the integration between sessions that are taught in different parts of the curriculum. Horizontal integration is that which occurs between specific fields of knowledge, such as between biochemistry and medicine when carrying out a urine dip test to diagnose a urinary tract infection. Whilst the FP teaching programme at an individual teaching hospital trust may plan for vertical integration, in practice, it may be that clinicians who are not directly involved in the education of trainees deliver individual teaching sessions. As a result, the extent to vertical integration may depend on individual clinicians being aware of the curriculum they are helping deliver and the prior knowledge of the trainees. Therefore, whilst the FPC is planned to be integrative, it is difficult to assess how integrated it is in practice, at the point of delivery in an individual hospital.

## How does the FPC achieve its aim?

The aim of the FP is essentially to prepare new medical graduates to enter speciality or general practice training. This aim is achieved by acquisition of competencies which are divided into four sections which are similar to the four domains stated in the GMC’s Good Medical Practice (GMC, 2013). The sections are: professional behaviour and trust, communication, team working and leadership, clinical care and safety and quality (UKFPO, 2016). These domains contain 20 descriptors of competencies, which could be termed broad learning outcomes, that trainees are expected to meet in order to pass their ARCP.

The FPC places a great deal of emphasis on effective communication between trainees and patients. This is reflective of the high expectation that patients have of their doctors. According to Ipsos (2017) data, doctors are amongst one of the most trusted professions in the public’s view. This results in a great responsibility upon doctors to maintain this trust in order to enable patients to trust doctors with their confidential health information. However, it is important to note that this survey did not delineate the grade of doctor and so it may be inferred that all doctors regardless of grade are amongst the most trusted health professionals.

Dale et al. (2008) explored the communication skills amongst SHO junior doctors in 1990 and 2005. They found that SHOs were utilising better communications skills in the studied Accident and Emergency department in 2005 than compared to 1990. Whilst numerous medical workforce changes had occurred throughout this 15 year period, Dale et al. (2008) acknowledge that the increase in patient-centeredness had played a key role in this improvement. The FPC was introduced between this period, and with the increased emphasis on communication and the patient-doctor relationship, it could partially explain the improvement in patient-doctor interaction between some SHOs (specifically FY2 doctors) and patients. The emphasis on key GMC domains by the FPC early in a doctor’s postgraduate medical education journey may improve the professionalism of trainees to a sufficient level for them to enter further training and also may have been as a reaction to maintain the trust needed between all doctors and their patients.

## The hidden curriculum

The FPC requires trainees to act in accordance with ethical principles in order to be signed off as being competent in both FY1 and FY2 years. However, ethics and by extension professionalism, is not explicitly taught within the two years of the FP and may be left to be taught within the hidden curriculum.

It may be surmised that provision of good care is a pre-requisite to entering the FP or is a concept that is acquired as trainees progress through the FP. Through validated and reliable assessment methods, in both medical school and in the FP, poor quality care may be challenged and remediation required in order to progress to the next stage of training. The FPC encourages trainees to practice in accordance with guidance from the GMC’s Good Medical Practice (GMC, 2013). This document requires all doctors to provide a good standard of practice and care, however it does not define what qualifies as good or bad care and this may need clarification by trainers. The words of Good Medical Practice (GMC, 2013) may form part of the formal curriculum of the FP, however their interpretation and real world implications may be taught in the informal curriculum and/or hidden curriculum by senior colleagues.

The hidden curriculum is defined as a separate concept to the formal curriculum which is itself distinct from the informal curriculum as explained by Hafferty (1998). The formal curriculum is outlined by Howard et al. (2012) as the material that is taught to students in lectures and seminars, whereas the informal curriculum is the impromptu teaching that is delivered by those teachers who are not directly attached to the core faculty. These two parts of the curriculum may appear to encapsulate the majority of teaching within a curriculum. However, as Howard et al. (2012) go on to explain, whilst trainees may gain some benefit from a formalised ethics based teaching program, it appears that formal classroom based ethics based teaching is preferred less by trainees than more concrete clinical teaching. This negative perception may translate into poor feedback to program administrators and so may inadvertently relegate ethics teaching to either the informal or hidden curriculum. The informal curriculum was examined by Hundert, Hafferty and Christakis (1996) and found to be the teaching that occurs around the formalised teaching, such as the instruction received by trainees by seniors just after a lecture has finished. In addition, the informal teaching may include the peer to peer teaching that occurs by senior trainees to their junior counterparts. Hundert, Hafferty and Christakis (1996) encourage using the informal curriculum to supplement the formal curriculum in ethics based teaching. They do this by encouraging learners to discuss such topics outside of the lecture theatre, in more informal settings such as oncall rooms or doctors’ offices on the wards.

The formal and informal curricula and the discussion around them appears to account for the majority of professionalism teaching that is received by medical students. However as Howard et al. (2012) explain, the hidden curriculum is the knowledge which cannot be articulated in some form of formal or informal instruction method. As a result, it may be that students learn professionalism from their seniors, such as professors at medical school or consultants when students are on their placements. It is pertinent to note that the hiddenness of the hidden curriculum is with regards to its implicit nature rather than any hidden agenda as explained by Ssebunnya (2013).

The hidden curriculum has been attributed by MacLeod (2014) as being the vehicle through which students acquire professional values within the umbrella of medical education. MacLeod (2014) describes hidden curricula as being the area where concepts are grouped together which may not be explicitly taught to students. This implies that the hidden curriculum is a nebulous concept and as a result may be defined differently by individual medical educators. This variation in definitions may affect the delivery of the hidden curriculum and by extension the sections of the overall curriculum which interact with it.

As FP trainees are to some extent a product of the medical schools from which they graduate, it may be relevant to briefly explore how values are taught to medical students. Ssebunnya (2013) argues that the shift towards scientific and technology based undergraduate medical education, due to a pursuit towards funding associated to research, has hampered the development of self-reflective medical school graduates. This is slightly alarming as a key part of the FPC focuses on reflective practice, with evidence of this required in the e-portfolio and therefore needed to complete the FP. Ssebunnya (2013) reasons that ethical principles, and by extension professional values, cannot be taught, rather acquired through observation at medical school through the hidden curriculum. Therefore, the hidden curriculum can be thought of as the vehicle through which the culture of a medical school is transmitted to a medical student. This can be extrapolated further into postgraduate medical education and therefore may imply that the hidden curriculum is the way in which medical values are transmitted to a trainee.

Whilst the FPC is currently regulated centrally by a distinct body, the United Kingdom Foundation Programme Office (UKFPO), the delivery of it is dependent upon local trusts and hospitals. As a result, the culture that is transmitted to trainees as they progress through the FP through the hidden curriculum is dependent upon supervisors and consultants acting as role models. Goldie (2000) argues a number of ways to address the hiddenness of the hidden curriculum. One of these ways is by encouraging good role-modelling as this has been shown by Goldie (2000) to have a more profound impact on learners than a formal ethics based teaching programme. This may be due to learners observing ethics in practice rather than learning the theory of medical ethics in a plenary session. Through replication of the behaviours of their role models trainees can demonstrate the “shows” how part of Miller’s pyramid (Miller, 1990) demonstrating that they have acquired and can demonstrate said behaviours. This experiential learning is thereby reliant on all trainees having good role models in order to have consistent ethics based teaching across every trust that has FP trainees. This may be difficult to ensure due to a limited number of supervisors, limited resources and time limitations. As a result, it may be an admirable goal to address the hiddenness of the hidden curriculum by having positive, self-reflective role models who show how to be an effective and reflective doctor. However, due to limitations on finding such supervisors, it may be the hidden curriculum is needed to some degree in order to teach ethical values to trainees as they progress through the FP.

## What are the assessment strategies of the FPC?

There are a number of assessment methods to assess a trainee’s clinical acumen in the FP. These include supervised learning events, which are effectively formative workplace-based assessments, such as the case-based discussion (CBD). The CBD is an assessment tool which encourages trainees to reflect upon a case that they have experienced in clinical practice and allows them to note any learning points that they have self-identified to discuss with their assessor. The assessor reviews the CBD and makes comments based upon the trainee’s entry in order to help guide their learning. However, the effectiveness of the CBD tool is variable and can depend upon the degree to which the trainee is willing to reflect upon their performance and the quality of the feedback given by trainers. Jyothirmayi (2012) explored the effectiveness of CBDs as a teaching tool amongst senior trainees and their consultant supervisors and found that trainees felt it was a useful tool to discuss ideas such as communication skills. However, given that there is a minimum number of CBDs needed to pass the ARCP for each Foundation Year, it may be that the trainees view them as hoops that they need to jump through for evidence on their e-portfolio in order to proceed to the next stage of their training. The theory of a portfolio being a means to an end, namely passing the ARCP, is supported by qualitative research by Halder, Subramanian and Longson (2012), who found that the top priority for trainees regarding portfolio use was to pass their ARCP. However, this same piece of research demonstrated that the second priority for trainees using the portfolio system of assessment was its use as a learning tool. This is somewhat reassuring as it shows that the e-portfolio is being used as evidence of trainees meeting the competencies that are detailed in the FPC.

The trainer’s report forms a crucial part of a trainee’s assessment and through it supervisors are able to give feedback in order to allow the trainee to improve. However, as trainees move placements, and as result move wards (usually every four months), there is a break in continuity of clinical supervision of a Foundation Year trainee. The FPC does require a trainee to have an educational supervisor for each year. However, in practice interaction between an educational supervisor and their trainee occurs far less than interaction between a trainee and their clinical supervisor. The discontinuity between in clinical supervision can also occur within a four-month placement due to differing shift patterns and so this can limit regular contact between a clinical supervisor and their trainee. The effect of the limitation in regular trainee-supervisor contact on assessments, like those mentioned above, was explored by Cheung et al. (2017). They state that the irregular contact between clinical supervisors and trainees may inhibit supervisors from giving negative criticism. This reluctance to give negative feedback is compounded by a presumption by supervisors that previous supervisor reports were satisfactory, further reducing the likelihood of a negative supervisor report being given. As a result, trainees may fall into a false sense of security regarding their level of clinical competence and hinder improvement of their clinical acumen. Cheung et al. (2017) go on to state that the quality of workplace-based assessments depends on the quality and depth of the educational relationship between supervisor and trainee, termed the educational alliance. It may be difficult to determine the exact effect of the strength of this alliance on the electronic written assessments that are required by the FPC.

## What are the methods of quality assurance of the FPC?

There are many methods of curricular control and refinement that exist in order to ensure that a curriculum is appropriate for the environment in which it is being taught. As part of satisfactory sign off in the ARCP, trainees are required to complete feedback forms on the placements that they have rotated through as a means of evaluation. This evaluation tool can be used as a quality management tool to allow for refinement in the delivery of the FPC. It asks, amongst other factors, the view of trainees on scheduled teaching and support in a training post. However, as Higgins, Cavendish and Gregory (2006) report, teaching sessions involving Foundation Year trainees can be poorly attended, due to service provision requirements. They state that this can negatively affect the delivery of the FPC. It is difficult to see how this can be mitigated centrally by the UKFPO as there may be local problems with service provision which may require a local solution in order to improve the training environment of trainees.

The FPC is no exception to regular revision by stakeholders, such as the regulator of doctors in the UK, the GMC. The GMC regularly produce guidance for standards in postgraduate medical education and these recommendations are intended to improve the quality of postgraduate medical education. The GMC requires all doctors in training to complete an annual National Training Survey. This is one of the methods that the GMC employ to quality assure GMC-approved training programmes, such as the FP. Data from this survey is shared with other stakeholders, such as Health Education England and Deaneries, who are responsible for delivery of the FPC. This enables collaboration to find a way of improving the delivery of the curriculum.

The FPC has many stakeholders and given the number of stakeholders this may lead to competing agendas with regards to the design of the curriculum. Wong (2012) explores how different stakeholders can collaborate in order to develop a postgraduate medical education curriculum in Canada using the Delphi technique. This technique is described in the literature as an effective tool to reach a consensus on the competencies required for a curriculum. Additionally, this technique avoids tensions that can occur with face-face meetings by collaborating electronically using forms and questionnaires. This appears to be an effective way of avoiding tensions that can occur with physical meetings to decide content for curricula, however the FPC does not explicitly state the method used to reach a consensus on which competencies to include. However, it does state in the bibliography, documents which have been used to shape the curriculum, such as the competency framework of the Academy of Medical Royal Colleges (UKFPO, 2016). The curriculum proposed by Wong (2012) is for an outpatient setting in Canada which is a different working environment than that of the FP in the UK. Therefore, whilst it does demonstrate an innovative approach to designing a curriculum through collaboration with many stakeholders, perhaps further research in this area is needed before it can be applied as a way of refining the FPC.

## Conclusion

The FPC was devised in response to a need to improve postgraduate medical education. The need to improve the educational climate for medical graduates was identified by the CMO of the UK at the time. Policy that was produced by the Department of Health recommended a two-year rotational programme with a spiral curriculum extending through both of these years as a way to streamline the early part of postgraduate medical education. The primary aim of the FPC was to enable doctors to develop their professional and clinical skills in order to proceed to the next stage of training. The mechanisms through which the curriculum is delivered may not always be through formal means and some of the more difficult topics may be explored though the hidden curriculum. The assessment of the FPC can be of variable quality depending upon the supervisor allocated to a specific trainee. The FPC has various quality control mechanisms, such as trainee evaluation tools, in order to identify problems and take steps to solve issues once they have been identified. In my opinion, the FPC has been shaped by various educational, social and political influences in order to produce an appropriate curriculum which allows trainees to develop as clinicians in order to enter the next stage of training.

## Take Home Messages

• The FP is a common bridge in the UK that medical graduates have to cross before entering into speciality training
• The FPC is a postgraduate medical curriculum that is designed to allow trainees to acquire the necessary knowledge skills and attitudes to progress into the next stage of their training
• The FPC can be thought of as an integrated spiral curriculum
• The FPC has various assessment strategies to determine if trainees have met the aims of the curriculum
• The hidden curriculum is an important aspect to consider when analysing a curriculum

## Notes On Contributors

Hassaan Waqar is a General Practice trainee in Birmingham in the United Kingdom with an interest in Medical Education and General Practice.

## Declarations

The author has declared that there are no conflicts of interest.

## Ethics Statement

No personal data which requires ethical approval was used at any stage in this manuscript.

## External Funding

This article has not had any External Funding
