# Supplementary material for: A Personal Critical Analysis of the Foundation Programme Curriculum
Source: MedEdPublish (2016). 2019 Apr 15;8:83. [Version 1] doi: 10.15694/mep.2019.000083.1 (PMC9926503; doi:10.15694/mep.2019.000083.1)
Supplement: Peer review history

# Peer review: Reviewer response for version 1

**DOI:** 10.21956/mep.19833.r29891

**Published:** 2019-09-16

**Authors:** Felix Silwimba

**Affiliations:** 1 University of Lusaka

This review has been migrated. The reviewer awarded 5 stars out of 5. This is an excellent write up on postgraduate medical education preparations. It in a way explains the role of a curriculum in continuing medial education and making medical doctors’ learners throughout their careers and beyond. Even though the write up describes medical education in the UK. I find it applicable in low income countries. I would equate the 2 years FP program to the ‘rural posting’ programs implemented in some low-income countries. Except that in such countries ‘rural posting’ is not officially listed as part of medical education leading to admission to a postgraduate course. Therefore, it is difficult to separate medical education from the practice of medicine since the formal, informal and hidden curriculum are inseparable. I thank the authors for their work.

# Peer review: Reviewer response for version 1

**DOI:** 10.21956/mep.19833.r29893

**Published:** 2019-09-17

**Authors:** David Bruce

**Affiliations:** 1 None

This review has been migrated. The reviewer awarded 3 stars out of 5. As a member of our local Deanery team who was involved at the start of Foundation training, I read and enjoyed this personal critical analysis of the Foundation Programme Curriculum (FPC). I think it will be of interest to medical students, Foundation doctors and those involved in the delivery and quality management of Foundation Programmes. The paper is wide ranging, covering the background to Foundation training in the UK, the curriculum aims, methods and assessments and quality assurance. The author is presenting a personal critical analysis using his recent experience as a Foundation trainee and educational theory and raises many interesting points. The author outlines the importance of a spiral FPC in allowing Foundation doctors to take on more responsibility as they progress in their training. I wondered if the difficulty in assessing the integrated nature of the FPC related to personal experience, as the 20 learning outcomes apply to the whole Foundation programme. Explaining how the learning outcomes are structured around the four GMC domains and highlighting the emphasis on patient centred care and good communication in Foundation training is helpful to the reader.The section on the hidden curriculum raises important points. The authors experience is that ethics and professionalism is not part of the formal or informal curriculum and is therefore left to the hidden curriculum. I suspect that this is not the case for many Foundation programmes across the UK. The importance of the hidden curriculum and the way norms and values are transmitted is highlighted. This section really makes the case for increased attention to the quality of the educational environment and need for positive role models. However, for many a Foundation doctors the chance to meet and discuss ethical dilemmas with peers and seniors and experience how decisions are made will happen as part of the informal FPC. The assessment strategies as described gives food for thought for all involved in teaching. The supervised learning event (SLE) should be a protected period of time where a trainee gets to sit down with a trainer, discuss a case, and receive feedback on how they are progressing. If trainees perceive SLEs as hoops to be jumped through, then the value of this tool is diminished. The importance of good clinical and educational supervision is well made. I would have been interested to hear the authors view of the value of the Foundation multi-source feedback (TAB). I thought there were a couple of areas where this paper could be improved. The summary paragraphs about why the Foundation programme and FPC were introduced could be revised. The Calman reforms of the 1990s established secondary care specialty training and the introduction of Summative Assessment set standards for GP training. This then left a gap – the SHO posts, where doctors were not part of a training programme. Although in this system many doctors received high quality training in these posts, some worked in service posts with little training, hence the need for Foundation training. The politics of the introduction of Foundation Programmes and changes to regulation are probably out with the scope of this paper. The final section on quality assurance of the FPC mixes the feedback given by trainees about their Foundation programmes and the role of the regulator in quality assurance of the Foundation programme and actual review and changes to the FPC.As stated, the author is to be commended for providing a paper that will be of interest to medical students, Foundation trainees and those involved in the delivery and quality management of Foundation training.

# Peer review: Reviewer response for version 1

**DOI:** 10.21956/mep.19833.r29892

**Published:** 2019-07-27

**Authors:** Ken Masters

**Affiliations:** 1 Sultan Qaboos University

This review has been migrated. The reviewer awarded 4 stars out of 5. The paper is a personal critical analysis of the Foundation Programme Curriculum in the UK. The author has done well to produce a detailed analysis of the curriculum, including its rationale, history and implications in execution. The author has also situated the narrative within the appropriate medical education literature to produce a succinct and balanced appraisal of the curriculum. While there are times when those readers who have a more detailed knowledge of the development and application of the curriculum may feel that some detail is obscured, and some reference to the “political” aspects could do with a little more elaboration, it is important to remember that everything cannot be covered in such a review. A good and useful read.

# Peer review: Reviewer response for version 1

**DOI:** 10.21956/mep.19833.r29890

**Published:** 2020-05-15

**Authors:** Sateesh Babu Arja

**Affiliations:** 1 Avalon University School of Medicine

This review has been migrated. The reviewer awarded 4 stars out of 5. I have no clue how the training programs work in the UK, and the procedures followed going into the specialty training there. However, I enjoyed reading this manuscript. The author started off with a small introduction and then explained the curricular models, assessments, and the quality control activities of the FP program. One of the most important concerns raised by the author is the delivery of the curriculum. The curriculum and syllabus is written well, but the delivery depends on the hospital and trust. I believe it is the same problem everywhere. The curriculum document might be written very well, but it depends on the effective delivery. Other concerns raised by the author are the hidden curriculum and the trainees' inability to attend teaching sessions due to service commitments. It is an excellent piece of reflective writing by a trainee who has the first-hand experience. This paper is useful for trainees and other stakeholders involved in quality control activities of the FP program.
